# Supplementary material for: Ultrafast acousto-optic mode conversion in optically birefringent ferroelectrics
Source: Nat Commun. 2016 Aug 5;7:12345. doi: 10.1038/ncomms12345 (PMC4980447; doi:10.1038/ncomms12345)
Supplement: Supplementary Information — Supplementary Figures 1-7, Supplementary Notes 1-4 and Supplementary References [file ncomms12345-s1.pdf]

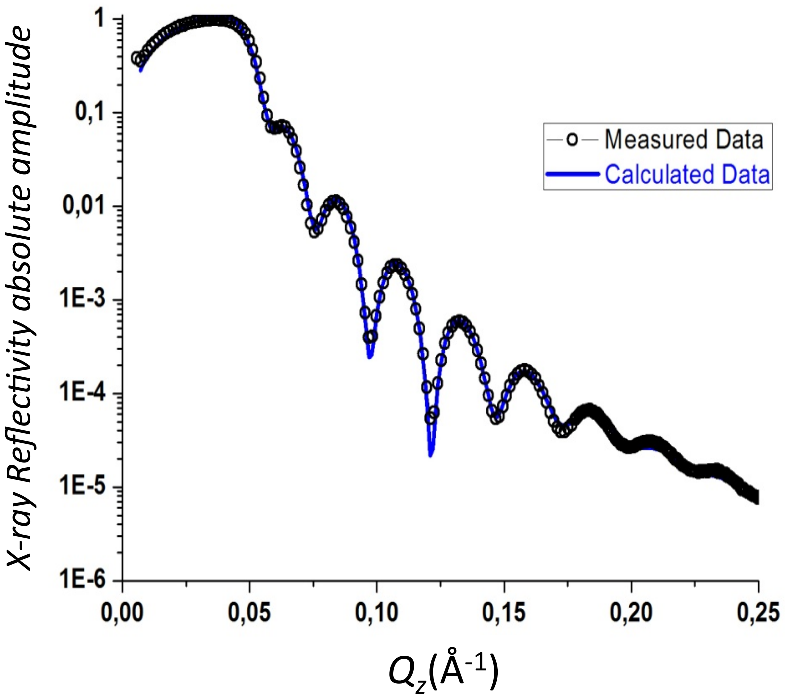

Supplementary Figure 1 - X-Ray reflectivity diagram obtained for a thin chromium film deposited on LNO. The Bragg oscillations permit to directly evaluate the chromium film thickness.

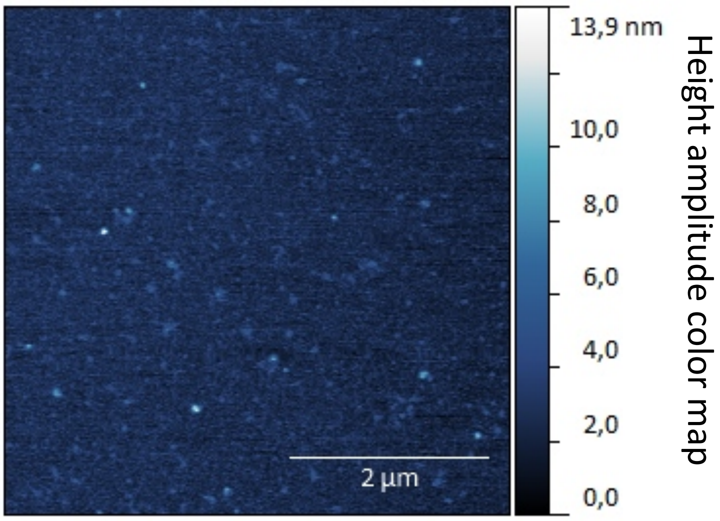

Supplementary Figure 2 - AFM picture (5x5 µm²) of chromium film. The topographical image has been obtained in tapping intermittent mode in air. The calculated RMS roughness is 0,680 nm.

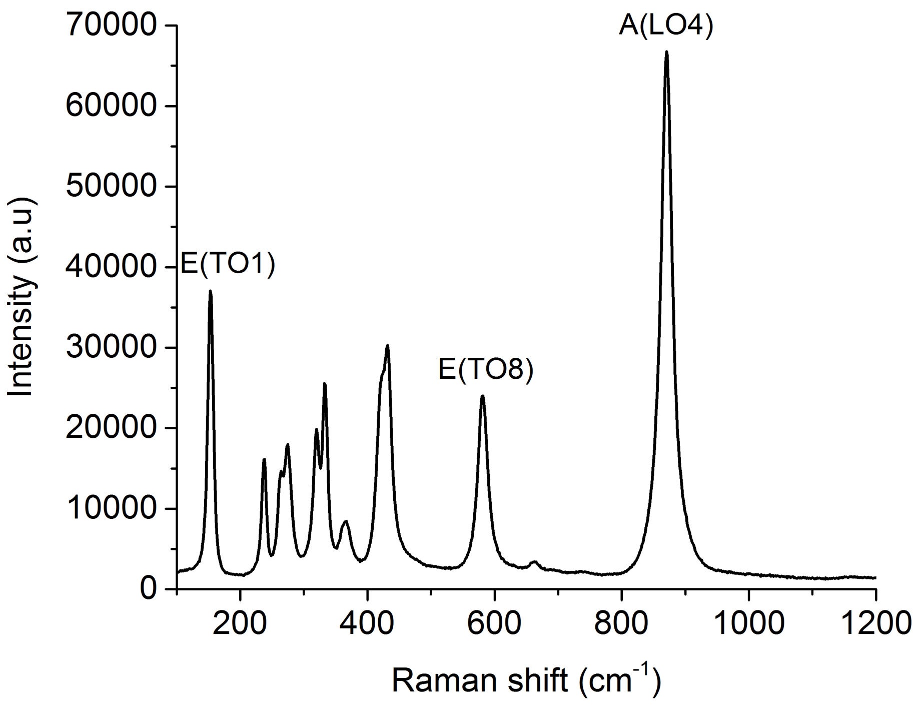

Supplementary Figure 3: Raman spectrum of stoichiometric Z-cut $LiNbO_3$ single crystals. All Raman peaks are assigned consistently Ref. [1].

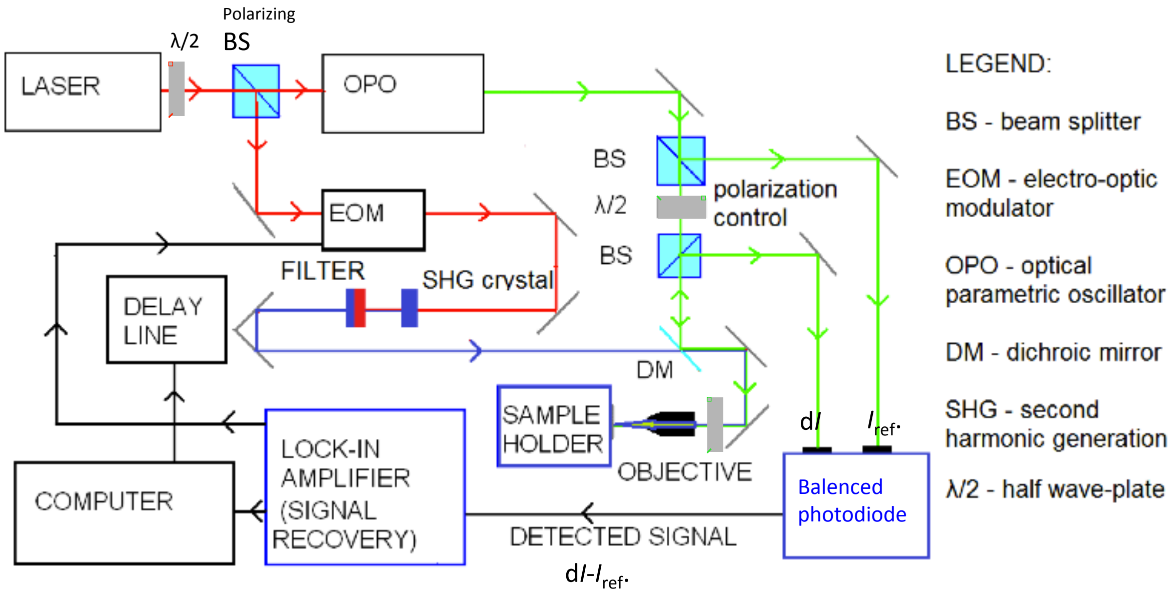

Supplementary Figure 4 : scheme of the two-color pump-probe setup.

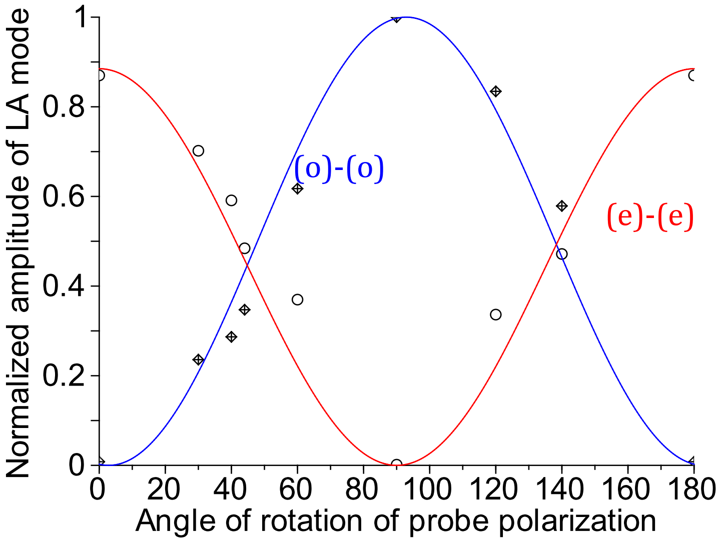

Supplementary Figure 5 : probe angle polarization dependence of the magnitude of the Brillouin process (o)-(o) and (e)-(e) obtained for calcite (CCO) crystal. The Brillouin peaks magnitude follows the cosinus function as shown by theoretical model presented in the manuscript (see Eqs. 5-6).

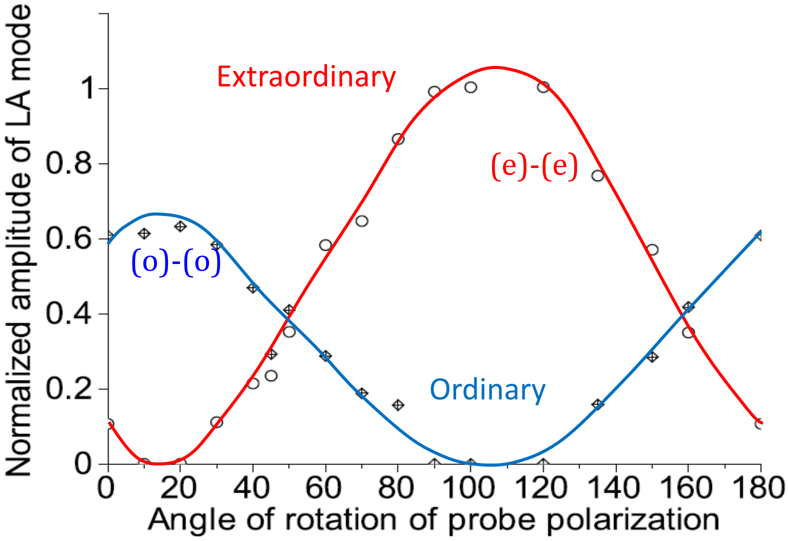

Supplementary Figure 6 : probe angle polarization dependence of the magnitude of the Brillouin process (o)-(o) and (e)-(e) obtained for Y-cut LNO crystal. The Brillouin peaks magnitude follow the cosinus function as shown by theoretical model presented in the manuscript (see Eqs. 5-6 in the manuscript).

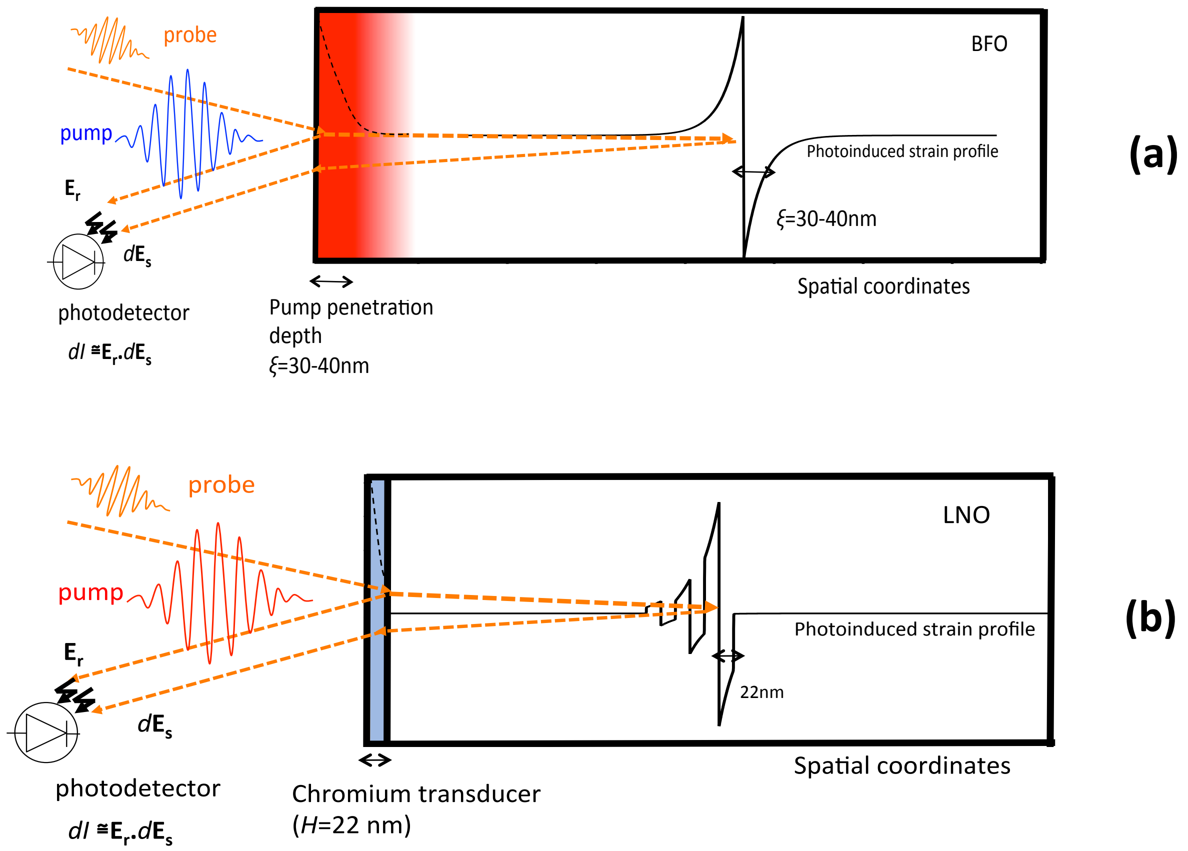

Supplementary Figure 7 : Simulation of the photoinduced strain profile in BFO (a) and in LNO(b). The photoinduced strain profile can be calculated based on the existing model in the literature [2,3,7] and thanks to the tabulated data of the optical penetration of the pump radiation in the absorbing medium. For BFO, the short acoustic pulse is governed by the pump penetration depth ξ at 400nm ξ=30-40 nm [4,5]. While for the case of Cr/LNO, the short acoustic pulse emitted in LNO is governed by the Chromium transducer thickness *H*.

Supplementary Note 1 : crystal quality and thermoelastic transducer deposition on $LiNbO_3$ (LNO) and Calcite (CCO) crystals.

The $LiNbO_3$ wafers and the calcite crystal were covered by a thin chromium film acting as a thermoelastic transducer following the traditional generation process in metals [2,3]. 20 nm thick chromium thin film was deposited with conventionnal physical vapor deposition (PVD) technique. The deposition was carried out in a Plassys ME300 evaporator system from a tungsten crucible containing chromium pellets at a pressure of about 10^{-7} mbar without heating the substrate. A quartz crystal microbalance was used for monitoring the chromium evaporation rate at 0,1nm/s. After deposition film thickness was measured with X-Ray reflectivity (XRR). Supplementary Figure 1 shows measured XRR data for our chromium thermoelastic transducer and calculated model obtained for a chromium film with the thickness of 22,8 nm.

Surface morphology was also characterized with an atomic force microscope (Keysight 5500) in intermittent mode in air. Root mean square (RMS) roughness of the film was calculated from

AFM pictures using Gwyddion software. AFM pictures were made on different areas of the films and RMS was always less than 1 nm. Supplementary Figure 2 shows an AFM picture measured on chromium film.

The $LiNbO_3$ (LNO) crystals (*X*, *Y* and *Z* cut) were provided by PI-KEM Ltd (http://pi-kem.co.uk/). These single crystals are of high quality and X-ray diffraction (not shown) and Raman spectroscopy characterizations were carried on to check that quality. Raman measurements of the stoichiometric $LiNbO_3$ single crystal were performed with a Renishaw spectrometer with a low-frequency cut-off at 100 cm^{-1}. The exciting laser line was 785 nm. As shown in Supplementary Figure 3, the values of the FWHM of the Raman peaks of the Z-cut LNO reveal the high quality of the crystal. Some Raman peaks are indexed consistently with the literature [1].

Supplementary Note 2 : ultrafast optical pump-probe measurements.

In Supplementary Figure 4 the scheme of the complete optical setup is given. Beam from aperture of femtosecond laser is first divided by polarizing beam splitter (polarizing BS) into two beams. Separation with desired intensities can be accomplished thanks to selection of polarization of light by half-plate. Red beam (830 nm) directed to EOM (electro-optic modulator) is called pump beam. Beam directed to OPO (optical parametric oscillator) and presented as green (for the case of probing with a wavelength of 560 nm for example) is called probe beam. Pump beam goes through barium borate (BBO) crystal for second harmonic generation (SHG). For the purpose of experiment light with wavelength 800nm or 830nm (depending on experimental configuration) was converted to 400nm, or 415nm, what corresponds to pump photon energies about 3.1eV and 2.99eV, respectively. The two pump photon quanta are larger than the $BiFeO_3$ band gap which leads to efficient generation of GHz coherent acoustic phonons. The second harmonic generation (SHG) is not necessary when the acoustic phonons are generated in the thin thermoelastic transducer (chromium film) that is deposited on the LNO or CCO crystals. In that latter case the laser harmonic (830 nm or 800 nm) is used.

The principle of the experiment is the following. The Pump pulse is absorbed in the near surface of the sample. Different particles (electrons) and quasi-particles (phonons) are excited and in this way leads to a modification of the optical properties of the material, such as reflectivity. This light-induced modification is detected thanks to a second laser beam called the probe beam. Pump and probe beams are superimposed in space thanks to a dichroic mirror (DM) and are both focused on the same point with a lens or microscope objective. Probe pulse is reflected from the sample and experiences change of reflectivity ΔR caused by the pump-induced transient state. Typical signal are shown in Figures 2-5 in the manuscript. That transient optical reflectivity ΔR is obtained thanks to the detection of the transient light probe intensity achieved with the photodiode (dI in the Supplementary Figure 6, dI is also shown in Figure 1(c) of the manuscript) and compared with reference signal (I_{ref}). This reference signal is subtracted from dI in order to filter out low frequency laser noise (balanced photodiode scheme). Since the laser induced probe light intensity change is small (relative variation of 10^{-4}-10^{-6} typically), the exciting pump beam is modulated in time with an electro-optic modulator (EOM, 80kHz) resulting in the modulation of the relevant detected signal dI. That signal is sent to the lock-in amplifier where it is demodulated. In order to sample the transient optical reflectivity with hundreds of femtosecond of resolution in time, an optical delay-line is used to convert the difference between the optical paths of pump and probe beams into a time delay. The long delay line permits to record signal up to 3ns which provides high frequency resolution when Fast Fourier Transform is performed on our time-resolved Brillouin signals. The probe angle dependence studies that permit to reveal all the Brillouin processes (See Figures 2-5 in the manuscript) have been realized thanks to the use of a suitable half-plate at the probe wavelength. The probe power has been systematically checked and readjusted to be constant for

each probe electric field angle. The pump power has also been checked to be constant in all experiments.

Photogeneration and photodetection of short acoustic pulse :

In our experiments (BFO, LNO and CCO), photoinduced coherent acoustic phonon pulse has a in-depth spatial extension controlled by the penetration of pump radiation in the absorbing medium.

The description of the profile has been already described by analytical theories [2, 3] which provide a clear insight in the phenomenon of Brillouin light scattering by coherent acoustic pulses. For the case of the BFO sample with the mechanically free surface, the acoustic pulses are photo-generated inside BFO. In order to represent the photoinduced profile we need the optical parameters that are tabulated in [4,5] and in which all the parameters (both temporal and spatial) of the acousto-optic interaction in BFO are given (for example the penetration lengths of the pump and probe laser pulses). The typical profile of photoinduced strain is given in Supplementary figure 7(a) and has the mathematical expression [2,3]:

$$\eta(z,t) = A \times sign(z - V_{LA,BFO}t) \times \exp\left(-\alpha\left|z - V_{LA,BFO}t\right|\right) \qquad \text{Eq.1}$$

where $\alpha = \frac{1}{\xi} = \frac{\lambda}{4\pi\kappa}$ is the optical absorption coefficient with κ the imaginary part of the refractive index of BFO at the pump wavelength λ. $V_{LA,BFO}$ is the longitudinal sound velocity in BFO. A is a parameter dependent on the physical mechanism of conversion of optical energy into acoustic energy (see recent review paper [6]).

The detailed analysis of the case of the metallic optoacoustic transducer (in our case it is the chromium layer deposited on the top of LNO crystal) can be found in [7]. The typical profile of emitted strain pulse in LNO after an optical excitation of the Cr layer is given in Supplementary figure 7(b). Because of reflections of photo-induced acoustic strain at chromium/LNO interface, the emitted acoustic pulse in LNO has a trail.

Concerning the spatial extension over which the acousto-optic mode-conversion occurs we can confidently say that it takes place within few nanometers as explained below. Actually the acousto-optic interaction/scattering does not take place either in the regions of homogeneous material or in the regions of the homogeneously strained material. Rather, the optical waves are scattered by the gradients of strain. Quantitatively, although in the classical theoretical formulas for the time-domain Brillouin scattering (picosecond ultrasonic interferometry [2,3], the technique which we use in our experiments, the signals are proportional to the spatial overlap integrals of electromagnetic field with acoustic strain, their integration by parts demonstrates straightforward that the interaction is localized at the strain gradients [8]. When the coherent acoustic pulses are photoexcited by light absorption near the mechanically free surface they commonly have two dominating consecutive phase of opposite polarity [9]. The duration of the transition front between two phases of the coherent acoustic pulse launched nearly ideally flat mechanically free surface grows with increasing duration of the pump laser pulses. In the case of the femtosecond pump laser pulses this duration is short and one could need to account for the possible roughness of the material surface. For example, even the roughness of 1 nm broadens the duration of the transition front in the coherent acoustic pulse to the duration of sound propagation across 2 nm depth, which exceeds the duration of pump laser pulses in our experiments. The duration of the transition front between the acoustic phases of opposite polarity could be potentially broadened by the acoustic wave attenuation. However there are no signs of any significant attenuation of acoustic waves at the spatial scale of several micrometers in our experiments (see Figs. 2 – 4). Thus in our experiments the spatial length of the transition front is expected to be of few nanometers and is much shorter than the durations of the leading

and trailing acoustic phases surrounding it, which are controlled by the penetration depth of light in BFO (about 30-40 nm [4,5,10]) or by the thickness of the metallic optoacoustic transducer deposited on LNO (22 nm, see Supplementary Figure 2). Thus the acoustic strain gradients in the transition front exceed at least by an order of magnitude the strain gradients in the leading and trailing fronts of the bipolar acoustic pulse (see supplementary Figure 7) and the mode conversion of the light pulses in our experiments is expected to take place at the spatial scale of few nanometers. This estimation is consistent with the theoretical treatment on a polarization control by using the refractive indices for a bulk state. It is worth noting that the recent experiments [11] have confirmed that the Brillouin scattering by the sharp front of the coherent acoustic pulse dominates over the light scattering by its other parts. The only limitation of the nanometric spatial localization of the mode conversion comes from the acoustic attenuation. Different contributions can smear the sharp photoinduced strain gradient like the surface roughness, the anharmonic acoustic attenuation and the pulse laser duration. In our case, the roughness of Cr layer for example is less than 1 nm which leads to a broadening of the leading front of around 2nm only. Moreover, there is no sign of significant acoustic attenuation in our high quality Brillouin signal (for the range 0-1ns) and finally a laser pulse as short as hundreds of femtosecond has a negligible contribution on the broadening of the sharpest photoinduced strain gradient compared for example to the effect of the roughness.
This information on the spatial scale of the light mode-conversion is important regarding possible future development of miniaturized acousto-optic devices.

Signal processing :
The signal extracted from the lock-in amplifier corresponds to the change of the optical reflectivity ΔR. The Fourier components of the Brillouin signals are obtained with Fast Fourier Transform (FFT) of the time-derivative of the transient optical reflectivity. As a main information, the quality of our signals (long-living Brillouin oscillation up to 3ns) provide us a very good frequency resolution (better than 1 GHz) which permit us to really distinguish all the Brillouin components without any advanced signal processing. In order to get nicer Brillouin signals, we have applied the traditional zero-padding method for a frequency interpolation.

Supplementary Note 3 : Probe polarization angle dependence in 45° single crystal of Calcite and Y-cut $LiNbO_3$ crystal.

The full probe polarization angle dependences are shown for the case of Calcite and *Y*-cut LNO in Supplementary Figures 4 and 5 respectively. Both of these configurations, as discussed in the manuscript, do not exhibit mode conversion and only conventional “normal” acousto-optic process, i.e. (o)-(o) and (e)-(e) are observed and exhibit a π periodic dependence as explained by Eqs. 5 and 6 in the manuscript.

Supplementary Note 4 :Mode conversion in calcite axis oriented at 45 degrees versus surface (rotation of photo-elestic tensor by 45 degrees)

In the following paragraph, we describe the theoretical model used to account for acousto-optic effect in inclined Calcite crystal (45°). In particular, we show that the acousto-optic mode conversion is expected to be small (negligible) due to intrinsic small values of photoelastic coefficients of Calcite (see Table 1 in the manuscript).

When optical axis is in plane and acoustic propagation along x-axis (propagation and detection normal to the surface) mode conversion of light incident along x-axis can be caused by nonzero

$\Delta\varepsilon_{yz}=\Delta\varepsilon_{zy}$. We assume the plane acoustic waves propagating along x therefore $\frac{\partial}{\partial z}=\frac{\partial}{\partial y}=0$ and in principle we have only 3 possible displacement gradients $\frac{\partial u_x}{\partial x},\frac{\partial u_y}{\partial x},\frac{\partial u_z}{\partial x}$ contributing to the strain. Then

$$\Delta\varepsilon_{yz}=P_{41}\frac{\partial u_x}{\partial x}+P_{45}\frac{\partial u_z}{\partial x}+P_{46}\frac{\partial u_y}{\partial x} \qquad \text{Eq. 2}$$

Where: P_{41}, P_{45} and P_{46} are the components of the photo-elastic tensor in the experimental coordination frame (see coordinate frame in Figure 4(a) of the manuscript for example).
When optical axis of the crystal is in surface plane coefficients P_{45} and P_{46} for crystallographic structure of LNO, BFO and calcite are equal to 0. So in this case it is predicted only single possibility of mode conversion by $\frac{\partial u_x}{\partial x}$. When polarization is precisely in plane of the surface, only pure longitudinal (LA) mode is excited and $\frac{\partial u_x}{\partial x}$ is present only in this mode, so only LA mode can cause mode conversion.

$$\Delta\varepsilon_{yz}=P_{41}\frac{\partial u_x}{\partial x} \qquad \text{Eq. 3}$$

As shown in Table I of the manuscript, the coefficient P_{41} is much smaller in calcite (-0.038) than in LNO (0.155).

For rotation of optical axis by 45 degrees relative to y axis equation describing mode conversion takes form (see details of calculation below):

$$\Delta\varepsilon_{yz}=\frac{P_{41}-P_{14}}{2\sqrt{2}}\frac{\partial u_x}{\partial x}+\frac{P_{14}}{2\sqrt{2}}\frac{\partial u_z}{\partial x}-\frac{1}{2}\left[P_{44}-\frac{P_{11}-P_{12}}{2}\right]\frac{\partial u_y}{\partial x} \qquad \text{Eq. 4}$$

In this case rotation of light polarization is possible by all 3 displacements gradients. In particular, if we still assume that one of quasi shear modes is not generated by symmetry principles (u_y=0 and $\frac{\partial u_y}{\partial x}$=0), there will be still potential possibility to rotate polarization by both components of both QLA and QTA waves. So if mode conversion is not observed in calcite by QLA wave, this could be because coefficient $\frac{P_{41}-P_{14}}{2\sqrt{2}}$ and transverse component of QLA wave is small. According to article of Nelson et al 1972 [12], coefficients P_{41} and P_{14} for calcite are more than 10 times smaller than other photo elastic coefficients, so absence of mode conversion detection is most probably caused by smallness of these coefficients.

Details of Calculation of photo elastic coefficients in rotated system.

General formula for elements of photo elastic matrix:

$$P'_{HK}=M_{HI}M_{KJ}P_{IJ} \qquad \text{Eq. 5}$$

Where matrix M is a matrix of rotation in given direction and in given angle
Therefore formula for element P_{41} will be:

$$P'_{41}=M_{4I}M_{1J}P_{IJ}$$
$$P'_{41}=M_{41}M_{1J}P_{IJ}+M_{42}M_{1J}P_{IJ}+M_{43}M_{1J}P_{IJ}+M_{44}M_{1J}P_{IJ}+M_{45}M_{1J}P_{IJ}+M_{46}M_{1J}P_{IJ} \qquad \text{Eq. 6}$$

Matrix of photo elastic coefficients for BFO and calcite has the form:

$$p_{ij} = \begin{bmatrix} p_{11} & p_{12} & p_{13} & p_{14} & 0 & 0 \\ p_{12} & p_{11} & p_{13} & -p_{14} & 0 & 0 \\ p_{31} & p_{31} & p_{33} & 0 & 0 & 0 \\ p_{41} & -p_{41} & 0 & p_{44} & 0 & 0 \\ 0 & 0 & 0 & 0 & p_{44} & p_{11} \\ 0 & 0 & 0 & 0 & P_{11} & p_{66} \end{bmatrix} \quad \text{Eq. 7}$$

Matrix of rotation of the system around axis y has the form:

$$M = \begin{bmatrix} cos^2\eta & 0 & sin^2\eta & 0 & -sin2\eta & 0 \\ 0 & 1 & 0 & 0 & 0 & 0 \\ sin^2\eta & 0 & cos^2\eta & 0 & sin2\eta & 0 \\ 0 & 0 & 0 & cos\eta & 0 & sin\eta \\ \frac{sin2\eta}{2} & 0 & -\frac{sin2\eta}{2} & 0 & cos2\eta & 0 \\ 0 & 0 & 0 & -sin\eta & 0 & cos\eta \end{bmatrix} \quad \text{Eq. 8}$$

Therefore for rotation angle equal to 45^0 it will takes values:

$$M = \begin{bmatrix} 1/2 & 0 & 1/2 & 0 & -1/2 & 0 \\ 0 & 1 & 0 & 0 & 0 & 0 \\ 1/2 & 0 & 1/2 & 0 & 1/2 & 0 \\ 0 & 0 & 0 & 1/\sqrt{2} & 0 & 1/\sqrt{2} \\ 1/2 & 0 & -1/2 & 0 & 0 & 0 \\ 0 & 0 & 0 & -1/\sqrt{2} & 0 & 1/\sqrt{2} \end{bmatrix} \quad \text{Eq. 9}$$

As it can be noticed coefficients: M_{41}, M_{42}, M_{43}, M_{45} are equal to 0 so equation for photo elastic coefficient P_{41} in new frame simplifies to:

$$P'_{41} = M_{44}M_{1J}P_{4J} + M_{46}M_{1J}P_{6J} = \\ = M_{44}[M_{11}P_{41} + M_{12}P_{42} + M_{13}P_{43} + M_{14}P_{44} + M_{15}P_{45} + M_{16}P_{46}] + M_{46}[M_{11}P_{61} \\ + M_{12}P_{62} + M_{13}P_{63} + M_{14}P_{64} + M_{15}P_{65} + M_{16}P_{66}]$$

Eq. 10

After taking into account zero coefficients of matrix M and P equation for desired coefficient simplifies to:

$$P'_{41} = \frac{1}{2\sqrt{2}}[P_{41} - P_{14}] \quad \text{Eq. 11}$$

Similarly with new coefficient P_{45}:

$$P'_{45} = M_{4I}M_{5J}P_{IJ} = M_{44}M_{5J}P_{4J} + M_{46}M_{5J}P_{6J} = \frac{1}{2\sqrt{2}}P_{41} \quad \text{Eq. 12}$$

And new coefficient P_{46}:

$$P'_{46} = M_{4I}M_{6J}P_{IJ} = M_{44}M_{6J}P_{4J} + M_{46}M_{6J}P_{6J} = \frac{1}{2}[-P_{44} + \frac{P_{11}-P_{12}}{2}] \quad \text{Eq. 13}$$

SupplementaryReferences :

[1] Margueron, S. *et al.* Resolved E-symmetry zone-centre phonons in LiTaO3 and LiNbO3. *J. Appl. Phys.* **111,** 104105 (2012).
[2] Thomsen, C., Grahn, H. T., Maris H. J. & Tauc J. Surface generation and detection of phonons by picosecond light pulses. *Phys. Rev. B* 34, 4129-4138 (1986).
[3] Thomsen C., Grahn H. T., Maris H. J., Tauc J. Picosecond interferometric technique for study of phonons in the Brillouin frequency range. *Opt. Commun*. 60, 55-58 (1986).
[4] Kumar A. et al., Linear and nonlinear optical properties of $BiFeO_3$. *Appl. Phys. Lett.* 92 121915 (2008).
[5] Zelezny V., Chvostova D., Pajasova L., Vrejoiu I., Alexe M. Optical properties of epitaxial $BiFeO_3$ thin film. *App. Phys. A* 100, 1217-1220 (2010).
[6] Ruello P., Gusev V. Physical mechanisms of coherent acoustic phonons generation by ultrafast laser action, *Ultrasonics,* 56, 21-35 (2015).
[7] Lin N.-N., Stoner R. J., Maris H. J., Tauc J. Phonon attenuation and velocity measurements in transparent materials by picosecond acoustic interferometry. *J. Appl. Phys.* 69, 3816-3822 (1991).
[8] V. E. Gusev V. E. Detection of nonlinear picosecond acoustic pulses by time-resolved Brillouin scattering. *J. Appl. Phys*. 116, 064907 (2014)
[9] Gusev V. and Karabutov A. Laser Optoacoustics, AIP, New York (1993).
[10] Schick, D. et al. Localized excited charge carriers generate ultrafast inhomogeneous strain in the multiferroic $BiFeO_3$. *Phys. Rev. Lett*. 112, 097602 (2014).
[11] Klieber C., Gusev V. E., Pezeril T., Nelson K. A. Nonlinear acoustics at GHz frequencies in a viscoelastic fragile glass former. *Phys. Rev. Lett*. 114, 065701 (2015).
[12] Nelson D. F., Lazay P. D., Lax M. Brillouin Scattering in Anisotropic Media: Calcite. *Phys. Rev. B* 6, 3109 (1972).
